# Near-Infrared Fluorescence Imaging Sensor with Laser Diffuser for Visualizing Photoimmunotherapy Effects under Endoscopy

**DOI:** 10.3390/s24051487

**Published:** 2024-02-25

**Authors:** Toshihiro Takamatsu, Hideki Tanaka, Tomonori Yano

**Affiliations:** 1 Exploratory Oncology Research & Clinical Trial Center, National Cancer Center, Kashiwa 277-8577, Chiba, Japan; toyano@east.ncc.go.jp; 2 Research Institute for Biomedical Sciences, Tokyo University of Science, Noda 278-0022, Chiba, Japan; 3 Department of Head and Neck Surgery, National Cancer Center Hospital East, Kashiwa 277-8577, Chiba, Japan; hidetana@east.ncc.go.jp; 4 Department of Gastroenterology and Endoscopy, National Cancer Center Hospital East, Kashiwa 277-8577, Chiba, Japan

**Keywords:** NIR fluorescence imaging, photosensitive substance, endoscope, in vivo, forceps port

## Abstract

The drug efficacy evaluation of tumor-selective photosensitive substances was expected to be enabled by imaging the fluorescence intensity in the tumor area. However, fluorescence observation is difficult during treatments that are performed during gastrointestinal endoscopy because of the challenges associated with including the fluorescence filter in the camera part. To address this issue, this study developed a device that integrates a narrow camera and a laser diffuser to enable fluorescence imaging through a forceps port. This device was employed to demonstrate that a laser diffuser with an NIR fluorescence imaging sensor could be delivered through a 3.2 mm diameter port. In addition, fluorescence images of Cetuximab-IR700 were successfully observed in two mice, and the fluorescence intensity confirmed that the fluorescence decayed within 330 s. This device is expected to have practical application as a tool to identify the optimal irradiation dose for tumor-selective photosensitive substances under endoscopy.

## 1. Introduction

Photodynamic therapy (PDT) and photoimmunotherapy (PIT) are treatment methods that involve the use of a tumor-selective photosensitive substance (PS) and a laser light with a wavelength that matches the absorption of the PS to induce a photochemical reaction and eliminate cancer cells [1,2]. In PDT, the PS and laser light are used to target and destroy tumor cells [3], while in PIT, the PS in combination with a tumor-specific antibody is used to target cancer cells, and the reaction to PIT leads to the activation of the immune system [4]. Although both PDT and PIT are effective treatment options for cancer, they have different mechanisms of action and may be more appropriate for specific types of cancer. In clinical practice, PDT using talaporphine sodium has been approved and implemented for the treatment of locally recurrent esophageal cancer, early-stage lung cancer, and primary malignant brain tumors following chemoradiotherapy or radiation therapy [5]. Currently, a phase III clinical trial is underway in patients with head and neck cancer utilizing cetuximab-IR700 (Cet-IR700) to target the epidermal growth factor receptor (EGFR) [6,7]. This drug has been approved in Japan for the treatment of unresectable locally advanced or locally recurrent head and neck cancer based on the outcomes of a phase I trial [8]. Investigator-initiated clinical trials are currently being conducted to evaluate the use of PIT for localized esophageal cancer and unresectable advanced or recurrent gastric cancer that are resistant to conventional therapies [9].

The successful delivery of PDT and PIT relies on the ability to activate the PS with sufficient light energy while selectively illuminating the tumor. However, excessive light energy can cause damage to normal tissues, resulting in serious adverse effects [10,11]. Conversely, insufficient light energy may reduce the treatment’s effectiveness. Therefore, it is critical to develop methods that accurately detect the extent of tumors and to determine the necessary and sufficient light irradiation dose to exert therapeutic effects. Previous research by Sato et al. reported that the fluorescence of Cet-IR700 can be observed by NIR light exposure [12,13]. Takashima et al. used a fluorescence imaging system (LIGHTVISION, SHIMADZU, Japan) to visualize the real-time drug accumulation of light-sensitive substances in tumors, and they demonstrated a correlation between the decay rate of the fluorescence intensity and the anti-tumor effect [14]. However, current endoscopes are unable to employ the configuration that is required for fluorescence observation when treatment is performed, especially in the digestive tract.

The proposed device, which integrates a narrow-diameter camera and laser diffuser, represents a novel technique for fluorescence imaging, introduced through the forceps port. This device has the potential to facilitate the administration of appropriate doses of fluorescence intensity while performing laser irradiation under general endoscopy, thereby optimizing PDT and PIT treatment. This study focused on the development of the proposed device and investigated the feasibility of visualizing PIT tumor accumulation and monitoring fluorescence intensity decay in vivo.

## 2. Materials and Methods

### 2.1. Development of Fluorescence Imaging Camera with Laser Diffuser

**Figure 1:**
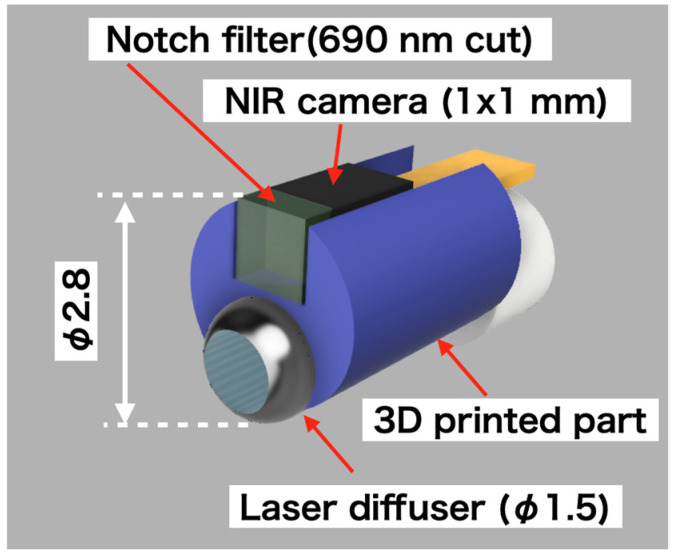
Three-dimensional design of fluorescence imaging camera with laser diffuser.

This study involved the design and construction of a device, shown in Figure 1, that could be inserted into an endoscope with a φ3.2 mm forceps port. The device was designed to emit laser light and acquire fluorescent images simultaneously. The sensor component of the structure was a 1 mm × 1 mm near-infrared camera (NIRBP, Opta Sensor, Nürnberg, Germany) equipped with a bandpass coating (813–878 nm transmission, OD4). The camera specifications were F2.7, FOV90°, 320 × 320 pixel, 10-bit depth, and a 10 ms exposure time as shown in Table 1. A 690 nm notch filter (672–698 nm block, OD6, Edmund Optics, NJ, USA) was attached to the tip of the camera to block high-intensity laser light. To prevent noise, dark images were prestored, and the acquired images were subtracted from the dark images.

A frontal diffuser (FD1; Medlight S.A., Ecublens, Switzerland) was used as the laser diffuser. The tip of the diffuser was covered with a Teflon tube (OD: 2 mm, ID: 1.5 mm), from which approximately 5 mm had been peeled from the top. The joint between the laser diffuser and the NIR camera was fabricated using a three-dimensional (3D) printer (XFAB 2000, DWS, Thiene, Italy) and a Therma284. The 2 m long flat cable for the camera and laser diffuser was connected using a heat-shrinkable tube (NR0502-003, Flonchemical Co., Ltd., Osaka, Japan).

### 2.2. Real-Time Fluorescence Imaging Using Test Sample by Developed System

To verify the fluorescence imaging functionality, negative and positive control liquids were analyzed using the developed system. Water was used as the negative control, whereas IRDye 700DX NHS Ester (IR700; Lincoln, NE, USA), diluted 50 times with PBS, served as the positive control. The excitation light source was a 690 nm laser (150 mW/cm^2^) from a laser irradiation device (MLL-III-690, Changchun New Industries Optoelectronics Technology Co., Changchun, Ltd., Changchun, China) that is used in PIT therapy. The probe head was positioned approximately 34 mm from the observation target. The acquired fluorescence image was black and white; thus, it was assigned a green color in the color image.

### 2.3. Animal Model

To obtain tumor-bearing mice, the A431 cell line was obtained from the American Type Culture Collection (Manassas, VA, USA). The cell line was cultured in Dulbecco’s modified Eagle’s medium (DMEM) (FUJIFILM Wako Pure Chemical Corporation, Osaka, Japan) supplemented with 10% fetal bovine serum (FBS, Thermo Fisher Scientific, Waltham, MA, USA) and 1% penicillin–streptomycin–amphotericin B suspension (FUJIFILM Wako Pure Chemical Corporation, Osaka, Japan). The cells were maintained at 37 °C and 5% CO_2_ in a humidified incubator. Two female BALB/c-nu nude mice at 6 weeks of age (Charles River Laboratories Japan, Inc., Yokohama, Japan) were implanted with 3.5 × 10^6^ A431 cells in the right waist. The tumor volume was calculated using the following equation: TV = (L × W^2^)/2, where L and W are the length and width of the tumor under the skin, respectively [15]. All the procedures and protocols were approved by the Animal Care and Use Committee of the National Cancer Center (K21-010-M04).

### 2.4. In Vivo Fluorescence Imaging Using Developed System

**Figure 2:**
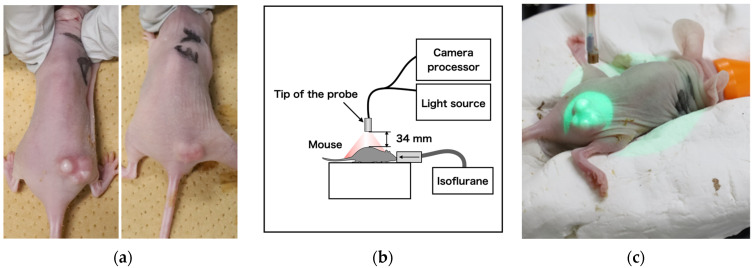
(**a**) Cet-IR700-dosed tumor-bearing mice, (**b**) Fluorescence imaging acquisition setup using mouse, and (**c**) laser diffuser irradiation method.

Cet-IR700 was used as an IR700-conjugated antibody for administration in tumor-bearing mice. Specifically, two mice with A431 tumors of approximately 100 mm^3^ were administered with 100 μg of Cet-IR700(Figure 2a), followed by laser irradiation for 24 h, and later for 330 s under anesthesia. The probe head was positioned approximately 34 mm from the center of the tumors as shown in Figure 2b, c. During this process, fluorescent images of the tumor region were captured. Pixel values were measured every 2 s to create a graph correlating light intensity with treatment time. After treatment, a halogen lamp (JCR15V150WS and LA-150UE, Hayashi-Repic Co., Ltd., Tokyo, Japan) with a light guide attached to an 850 nm long pass filter (FELH0850, Thorlabs, Inc., NJ, USA) was used, and the tip of the light guide was positioned above the mice to avoid halation. Image acquisition was performed to merge the fluorescence image with the overall image of the mouse. To enhance the hue, the intensity of the fluorescence image was doubled, assigned to green, and integrated into the image irradiated with a halogen lamp.

## 3. Results

**Figure 3:**
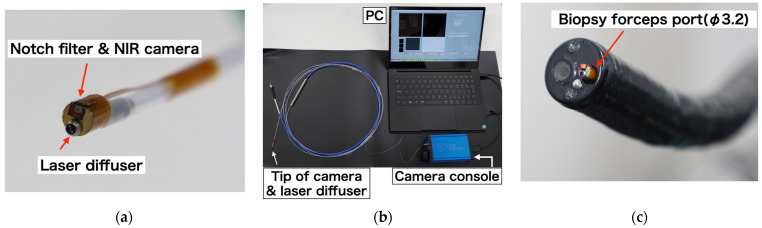
(**a**) Laser irradiation device with a built-in near-infrared fluorescence imaging sensor, (**b**) overview of fluorescence imaging acquisition system, and (**c**) photograph of an endoscope inserted into a 3.2 mm diameter forceps port.

We successfully developed a device that integrates a narrow camera and a laser diffuser, as depicted in Figure 3a. An overview of the developed device is shown in Figure 3b. This device has the ability to reduce the excitation wavelength of the laser and has a diameter of less than 3 mm, allowing it to be inserted into a 3.2 mm diameter channel using the forceps port on an endoscope, as illustrated in Figure 3c.

**Figure 4:**
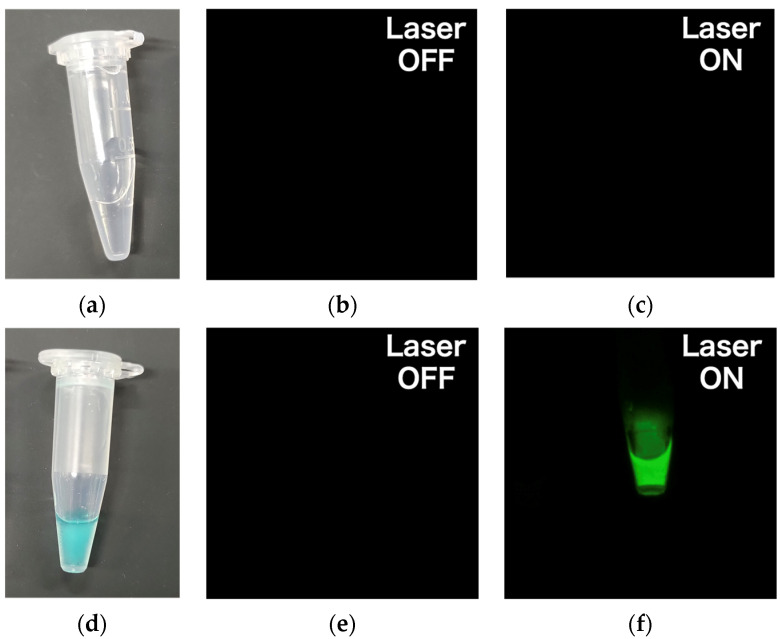
(**a**) A 1 mL tube containing water as a negative control, (**b**) image of the tube containing water taken by the fluorescence sensor before laser irradiation, (**c**) image of the tube containing water taken by the fluorescence sensor during laser irradiation, (**d**) 1 mL tube containing IR700 solution as a positive control, (**e**) image of the tube containing IR700 solution imaged by the fluorescence sensor before laser irradiation, and (**f**) image of the tube containing IR700 solution imaged by the fluorescence sensor during laser irradiation.

This device was used to image phantoms containing water and the IR700 solution, as shown in Figure 4a,d. As shown in Figure 4b,c, no fluorescence was detected in the water sample, whereas fluorescence was observed in the IR700 tube when exposed to the laser, as shown in Figure 4e,f. This confirmed that the laser wavelength was blocked by the filter, and only the fluorescence wavelength was captured.

**Figure 5:**
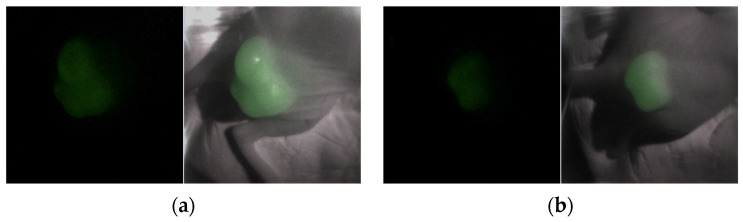

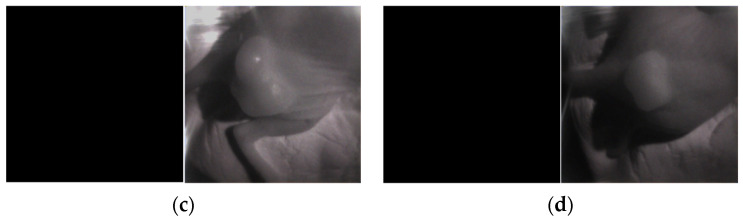
(**a**,**b**) Merged fluorescent images of each mouse immediately after laser irradiation and images irradiated by halogen lamps. (**c**,**d**) Merged fluorescent images of each mouse 330 s after laser irradiation and images irradiated by halogen lamps.

**Figure 6:**
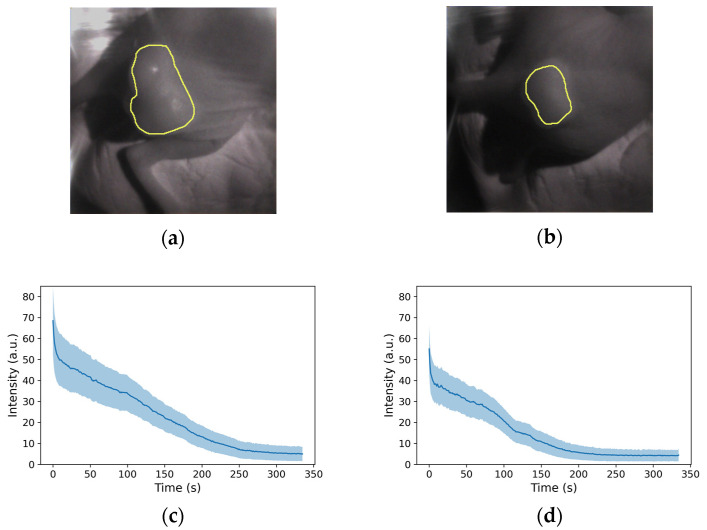
(**a**,**b**) Boundaries(yellow line) of pixels taken to average intensity. (**c**,**d**) Relationship between laser irradiation time and decay of fluorescence intensity for each mouse.

Fluorescence was detected in two mice (A431) who were administered with cetuximab-IR700 and exposed to a 690 nm laser (150 mW/cm^2^). As depicted on the left side of Figure 5a,b, fluorescence was evident immediately after irradiation in both cases. When merged on an image that was illuminated by a halogen lamp, as shown on the right side of Figure 5a,b, the fluorescence of the tumor region was visible. After 330 s of irradiation, as shown in Figure 5c,d, the fluorescence diminished and disappeared. To quantify the fluorescence intensity over time, the tumor area was designated as the region of interest, as shown in Figure 6a,b. The number of pixels was 10,105 and 4557 px, respectively. The mean and standard deviations are plotted in Figure 6c,d, respectively, and it was observed that in both mice, fluorescence rapidly declined immediately after irradiation; the decay of the fluorescence intensity reached equilibrium after approximately 300 and 250 s, respectively.

## 4. Discussion

Takashima et al. conducted a study using nine mice in a fluorescence imaging experiment and found that the fluorescence decayed rapidly after irradiation and reached equilibrium between 150 and 330 s, maximizing the anti-tumor effect [14]. As the present results exhibited similar behavior, the obtained fluorescence images were considered reliable. Previous research has demonstrated a relationship between the required laser dose and fluorescence intensity, emphasizing the significance of visualizing the therapeutic effects. Ongoing observational studies on head and neck surgery aim to confirm fluorescence attenuation in humans (UMIN000052240). Moreover, interest extends to endoscopic observation, with potential applications in the medical field. In this study, the feasibility of observing drug accumulation in tumors under endoscopy and identifying a sufficient irradiation time for PS was demonstrated using the developed device. This device has the potential for application not only for PIT but also for the fluorescence imaging of PDT and indocyanine green (ICG) under endoscopy.

In addition to the imaging system that was used in this study, other methods to obtain fluorescent images under endoscopy can be considered. For example, there are endoscopes with a notch filter-coated sensor that cut laser wavelengths and image transmission using bundled fiber [16,17]. For coating conventional endoscopes with notch filters, it is necessary to design the coating to match the fluorescent wavelengths for various PSs; however, this is difficult to achieve due to the high cost of developing endoscopes that are optimized to individual applications. As for the image transmission method using bundled fiber, fluorescent images can be easily obtained to adopt an optimal bandpass filter. However, since ordinary endoscopes are equipped with only one forceps port, it is necessary to integrate the laser diffuser. Consequently, the stiffness of the integrated fibers adversely affects the operability of the endoscope. Moreover, manufacturing an image fiber with an objective lens attached to the bundle fiber is expensive. By contrast, laser diffusers are disposable. Thus, an integrated configuration is difficult to achieve from an economic standpoint.

The advantage of the method developed in this study based on a small image sensor is that the sensor cable is flexible, and its stiffness is almost the same as that of a conventional laser diffuser; the sensor can also be configured to be disposable, as in the SpyScope™ [18]. In addition to PIT and PDT, there have also been reports on the availability of fluorescence wavelengths in the NIR region [19,20,21]. It is suggested that individual coatings of fluorescence wavelengths will likely expand the applications of the system.

To introduce the device into clinical practice, improvements in visibility and usability include a wider viewing angle, a new optical filter design, and a structure that allows the tip of the device to be curved. Although adopting a wide-angle lens may result in a reduction in resolution, our results indicate that tumor volumes of 100 mm^3^ or more can still be effectively identified as fluorescent regions, even with a resolution decrease of up to two or three times. The typical field of view of an endoscope is approximately 140°, whereas the camera used in this study had a field of view of 90°. Thus, by integrating a wide-angle objective lens, the observations can be compared with an endoscopic view.

With respect to the optical filter section, only cameras that are coated with a bandpass filter of OD4 are commercially available. Consequently, a notch filter of OD6 was added to this device. However, notch filters may be unnecessary when using an order-made image sensor coated with a higher-optical-density filter. Regarding the need for a curved structure, it is difficult for the frontal diffuser to irradiate in the lateral direction with only endoscopic curvature; however, it is difficult to install a fluorescence imaging sensor with a cylindrical diffuser. Therefore, the use of a curved tip device such as the SpyScope™ biliary speculum is expected to enable lateral irradiation [18]. This structure could be achieved by making the laser diffuser thinner and softer. One of the potential limitations of the proposed imaging method is the difficulty in registering the observed fluorescence onto the endoscopic image. This issue may be resolved by image registration processing, such as an affine transformation, before endoscopy.

The proposed configuration can be deployed as a single-use device, because a disposable camera is mounted on a laser diffuser, which is disposable in clinical use. Moreover, because there is no need to add a new filter for endoscopes, and the PS effect can be visualized regardless of the manufacturer of the endoscope, the system is considered economically viable and is expected to be applied clinically in the near future.

## 5. Conclusions

In this study, we successfully developed a laser irradiation device with a built-in near-infrared fluorescence imaging sensor that can be introduced into the forceps opening of an endoscope. The imaging sensor transmitted only the fluorescence wavelength, and it was confirmed that the fluorescence of Cet-IR700 that accumulated in the tumor could be visualized. The decay of the fluorescence intensity by laser irradiation was measured using the developed device, and it was confirmed that the intensity decreased with the irradiation time and reached equilibrium at approximately 330 s. This behavior is similar to that observed in a previous study that used a fluorescent camera (LIGHTVISION) to visualize the fluorescence of PIT in mice. Thus, we show the visualization of the decay of PIT fluorescence under an endoscope in a clinical setting.

## Figures and Tables

**Table 1:** Specifications of the developed camera sensor.

Element	Specification
Camera dimensions	1.0 mm × 1.0 mm × 2.0 mm
Lens	F2.7 FOV90 deg
Cable	2 m
Pixel size	2.4 µm × 2.4 µm
Pixel number	320 × 320
Exposure time	10-ms
Gradation	10-bit
Bandpass coat	813–878 nm
OD, 300–787 nm, 908–1100 nm	≥4
OD, 672–698 nm	≥6

## Data Availability

If new results are reported using the data from this study, discussions with the supplier of the fluorescent reagents are required based on non-disclosure agreement. Therefore, please contact the corresponding author if you wish to use the data.
